# The Phenotypic Spectrum of 16p11.2 Recurrent Chromosomal Rearrangements

**DOI:** 10.3390/genes15081053

**Published:** 2024-08-10

**Authors:** Anastasios K. Mitrakos, Konstantina Kosma, Periklis Makrythanasis, Maria Tzetis

**Affiliations:** 1 Laboratory of Medical Genetics, Medical School, National and Kapodistrian University of Athens, St. Sophia Children’s Hospital, 11527 Athens, Greece; 2 University Research Institute for the Study and Treatment of Genetic and Malignant Disorders of Childhood, 11527 Athens, Greece

**Keywords:** 16p11.2, copy number variants (CNVs), distal 16p11.2 deletion syndrome, proximal 16p11.2 deletion syndrome

## Abstract

The human 16p11.2 chromosomal region is rich in segmental duplications which mediate the formation of recurrent CNVs. CNVs affecting the 16p11.2 region are associated with an increased risk for developing neuropsychiatric disorders, including autism spectrum disorder (ASD), schizophrenia, and intellectual disability (ID), as well as abnormal body weight and head circumference and dysmorphic features, with marked phenotypic variability and reduced penetrance. CNVs affecting the 16p11.2 region mainly affect a distal interval of ~220 Kb, between Breakpoints 2 and 3 (BP2–BP3), and a proximal interval of ~593 Kb (BP4–BP5). Here, we report on 15 patients with recurrent 16p11.2 rearrangements that were identified among a cohort of 1600 patients (0.9%) with neurodevelopmental disorders. A total of 13 deletions and two duplications were identified, of which eight deletions included the proximal 16p11.2 region (BP4–BP5) and five included the distal 16p11.2 region (BP2–BP3). Of the two duplications that were identified, one affected the proximal and one the distal 16p11.2 region; however, both patients had additional CNVs contributing to phenotypic severity. The features observed and their severity varied greatly, even between patients within the same family. This article aims to further delineate the clinical spectrum of patients with 16p11.2 recurrent rearrangements in order to aid the counselling of patients and their families.

## 1. Introduction

The 16p11.2 chromosomal region is known to be susceptible to structural rearrangements as a result of the large number of segmental duplications that exist in this specific segment [1]. Such rearrangements can lead to the formation of recurrent Copy Number Variants (CNVs) of fixed sizes through the process of Non-Allelic Homologous Recombination (NAHR) [2].

CNVs affecting the 16p11.2 chromosomal region are associated with an increased risk for developing neurodevelopmental disorders, including autism spectrum disorder (ASD), schizophrenia, and intellectual disability (ID) [3], with additional features including obesity, dysmorphic features, and abnormal head size [4,5], all while exhibiting a very high degree of phenotypic variability and reduced penetrance.

**Figure 1:**
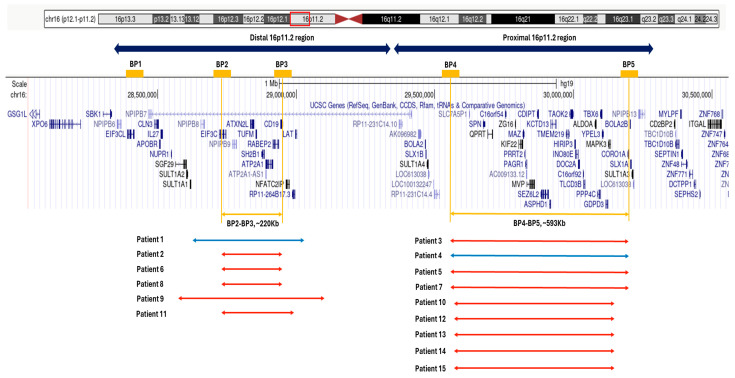
The 16p11.2 distal and proximal regions and the genes included in the region. Segmental duplications where the breakpoints for the rearrangements located are marked in orange (BP1–BP5). The approximate location of the rearrangements that were identified in our patients is also shown (blue: duplication, red: deletion). All coordinates are according to hg19 (GRCh37).

In humans, 16p11.2 CNVs affect mainly the distal interval of ~220 Kb, between Breakpoints 2 and 3 (BP2–BP3), and the proximal interval of ~593 Kb (BP4–BP5) [6]. Additional recurrent CNVs identified in the region include the distal 16p11.2 BP1–BP4 (~800 Kb) and BP1–BP3 (~550 Kb), as well as the distal–proximal BP1–BP5 (~1.7 Mb) [3] (Figure 1).

Both deletions and duplications of the proximal region (BP4–BP5) are classified as distinct syndromes in the Online Mendelian Inheritance in Man (OMIM) database (chromosome 16p11.2 deletion syndrome 593-Kb #611913 and chromosome 16p11.2 duplication syndrome #614671), with the main region affected spanning from ~29.6 to 30.2 Mb (hg19, GRCh37) from the telomere.

Deletion 16p11.2 syndrome is a contiguous-gene disorder associated with a characteristic, recognizable pattern of dysmorphic features, congenital anomalies, macrocephaly, developmental delay (DD), autism, hypotonia, and obesity. Patients with duplication of the 16p11.2 region exhibit some overlapping features with the deletion of this region, including ASD, and also present with mirror phenotypes of microcephaly and being underweight.

The distal region (BP2–BP3), with a size of 220 Kb, is also regularly affected by deletions, is associated with isolated severe early-onset obesity as well as developmental delay, and encompasses approximately nine genes, including the *SH2B1* gene (chromosome 16p11.2 deletion syndrome 220-Kb #613444).

Here we report on 15 patients with recurrent 16p11.2 rearrangements (13 deletions, two duplications) that were identified among 1600 patients with varying indications referred for chromosomal microarray analysis.

## 2. Materials and Methods

### 2.1. Patients

A total of 1600 patients with ID, DD, and/or ASD, with or without multiple congenital anomalies were referred for chromosomal microarray analysis to the Laboratory of Medical Genetics of the National and Kapodistrian University of Athens, between 2008 and 2023.

Out of these, 15 patients (0.9%) were found to carry a CNV affecting the 16p11.2 region.

### 2.2. Clinical Descriptions

#### 2.2.1. Patient 1

Patient 1 is a 5-month-old female patient born at term after an uneventful pregnancy, weighing 2470 gr (3rd centile, −1.82 SD). She was referred to the Laboratory of Medical Genetics of the National and Kapodistrian University of Athens for examination by a clinical geneticist. She had a height of 62 cm (30th centile, −0.53 SD), a weight of 6100 gr (23rd centile, −0.75 SD), and a head circumference of 43.5 cm (91st centile, +1.31 SD). She presented with central hypotonia, microcephaly, peripheral hypertonia, discoloration in the body and limbs, and dysmorphic features, which included small eye slits, a small nose, inverted flaps, slim lips, an arched palate, a short neck, brachydactyly, and a curly toe. She also had seizures since 1 month of age, which were successfully treated with oxcarbazepine, vigabatrin, and levetiracetam. She was able to eat only ground food. Metabolic screening and an abdomen ultrasound presented normal results. A brain MRI revealed dysplastic morphology in the right brain hemisphere with smaller dimensions and pachygyria. A heart ultrasound revealed patent foramen ovale and patent ductus arteriosus. Her mother is a carrier of a duplication in the X chromosome that includes the *PLP1* gene, associated with Pelizaeus–Merzbacher syndrome. The mother’s sister had a child with Sneddon’s syndrome presenting with a skin rash, vasculitis, and stroke, and who passed away at the age of 10.

#### 2.2.2. Patient 2

Patient 2 is a 1-year-old female born at term after a normal perinatal period, weighing 3000 gr (21st centile, −0.81 SD). Clinical examination revealed microcephaly, developmental delay, psychomotor delay, and strabismus. She was not able to walk nor talk and was able to sit without support at 11 months. She had a head circumference of 43.5 cm (11th centile, −1.23 SD) and a weight of 10 kg (66th centile, +0.42 SD). A brain MRI demonstrated signal abnormality in the periventricular white matter with lesions extended to the subcortical white matter, more prominent in T2-w and FLAIR images, indicative of a metabolic disorder; however, testing came out negative.

#### 2.2.3. Patient 3

Patient 3 is a 6-year-old boy, the 3rd child of healthy, non-consanguineous parents. He was born at date after an uneventful pregnancy, weighing 3100 gr (23rd centile, −0.74 SD), with a height of 49 cm (33rd centile, −0.44 SD) and a head circumference of 34 cm (20th centile, −0.83 SD). Clinical examination showed diffuse developmental disorder, developmental delay, psychomotor delay, obesity, bilateral cryptorchidism, and an arched palate. He walked at 18 months and talked at 30 months after attending logotherapy and ergotherapy since 4 months of age. Additionally, he presented with atopic dermatitis and recurrent infections of the respiratory system. A heart and abdomen ultrasound as well as neurological and ophthalmological examinations were normal, with good eye contact. Currently, he has a height of 122.3 cm (92nd centile, +1.39 SD), a weight of 46.8 kg (>97th centile), a head circumference of 56.6 cm (>97th centile), and a BMI of 31.29 kg/m^2^ (>97th centile).

#### 2.2.4. Patient 4

Patient 4 is a 6-month-old female patient, the first child of healthy, non-consanguineous parents. She presented with low muscle tone, a hypoplastic lower jawbone, low set ears, a sandal gap, increased distance between the nipples, macroglossia, a big forehead, hypertrichosis, deep-set eyes, and a low hairline. Additionally, she developed hypoglycemia during the second day of life. Metabolic screening produced a negative result. A heart ultrasound revealed congenital heart anomalies that included a ventricular septal defect, patent foramen ovale, and patent ductus arteriosus. A brain ultrasound and a subsequent MRI showed dilation of the lateral brain ventricles (ventriculomegaly). An abdomen ultrasound revealed hydronephrosis and renal cysts. Genetic testing for Beckwith–Wiedemann and Silver–Russel syndromes produced a negative result. The mother is a carrier of a balanced translocation between chromosomes 9 and 10 (46,XX,t(9;10)(p24;p13)).

#### 2.2.5. Patient 5

Patient 5 is a 15-year-old boy, born at 38 weeks after an uneventful pregnancy, weighing 2750 gr (10th centile, −1.30 SD). Clinical examination revealed brachydactyly of the upper and lower limbs, learning difficulties, mild dyslexia, congenital vertebral anomaly (hemivertebrae), advanced bone age, a club foot since 1 year, walking and talking at 1 year, scoliosis and lordosis since the age of 3, pelvic muscle stiffness, a hypoplastic left wing of the ilium, and short stature. He had no dysmorphic features and normal ears. He had slightly reduced growth hormone. A brain MRI produced a normal result.

#### 2.2.6. Patient 6

Patient 6 is a 5-year-old boy, born at term with a weight of 3140 gr (25th centile, −0.67 SD), a height of 52 cm (76th centile, +0.7 SD), and a head circumference of 33 cm (<1st centile, −14.39 SD). A prenatal ultrasound showed increased nuchal translucency, after which conventional cytogenetic analysis was performed on a chorionic villi sample, showing a normal karyotype (46XY). He presented with developmental delay, speech delay, stereotypic behavior, ASD, obsessions, and attention deficit, characterized by the use of non-verbal communication (signs) and single words only or just the first syllable of words. Receptive language was better than production. Assessment with Griffith’s Mental Development Scales (GMDS-R) and the Autism Diagnostic Observation Schedule (ADOS) showed a locomotor scale of 3 y 2 mo, eye and hand coordination of 3 y, a performance scale of 3 y 5 mo, and a hearing and language scale of 2 y 6 mo. He was able to sit at 6 months and walk at 10 months. He has been under logotherapy and ergotherapy since 18 months of age. A heart ultrasound, EEG, and brain CT produced normal results.

#### 2.2.7. Patient 7

Patient 7 is a 4-year-old boy, the second child of a twin pregnancy that occurred after IVF with the use of donor eggs. He was born at term with a weight of 1750gr (<1st centile, −2.9 SD), while his twin sister weighed 2350 gr (2nd centile, −2.05 SD). He presented with a diffuse developmental disorder and speech delay but had good perception. He had hypermetropia, a small nose, a small jaw, and a pointy chin. Additionally, he developed autoimmune neutropenia and was susceptible to infections. An abdomen ultrasound revealed mild enlargement of the liver and spleen.

#### 2.2.8. Patient 8

Patient 8 is a 4-year-old boy, born at 36 weeks, weighing 2550 gr (5th centile, −1.62 SD). He had a normal head circumference of 51.5 cm (77th centile) and presented with psychomotor delay and Attention Deficit Hyperactivity Disorder (ADHD). He was subjected to physiotherapy since 1 year of age and was able to walk at 2 years of age. He has been undergoing ergotherapy since 18 months of age. Heart and renal ultrasounds produced normal results.

#### 2.2.9. Patient 9

Patient 9 is a 3-year-old boy born prematurely at 28 weeks. He presented with intrauterine growth restriction (IUGR) weighing 740 gr (<1st centile, −4.5 SD) at birth, with a head circumference of 24 cm (<1st centile, −5.2 SD) and a height of 34cm (<1st centile, −6 SD). He remained in the neonatal ICU for 7 months with Cytomegalovirus (CMV) infection. He also developed retinopathy and anemia from prematurity. He does not speak and presents with a sensory disorder. A heart ultrasound showed patent ductus arteriosus and bronchopulmonary dysplasia. Clinical examination revealed cryptorchidism, hypospadias, and icterus. A brain MRI produced a normal result.

#### 2.2.10. Patient 10

Patient 10 is a 3-year-old boy, born at 40 weeks weighing 3540 gr (49th centile, −0.03 SD) with a height of 51 cm (62nd centile, +0.32 SD) and a head circumference of 35.8 cm (48th centile, −0.05 SD). He presented with a diffuse developmental disorder, psychomotor delay, iris coloboma, optical nerve coloboma, strabismus, autism, and seizures. He was able to sit at 6 months and walk at 14 months with support. He is not able to talk although he has normal hearing, shows fine motor skills impairment, and presents with problems with social interaction. A brain MRI and EEG produced normal results. A heart ultrasound revealed an atrial septal defect due to patent foramen ovale.

#### 2.2.11. Patient 11

Patient 11 is a 1-year-old boy born at 40 weeks after an uneventful pregnancy with a birth weight of 3115 gr (24th centile, −0.71 SD), a height of 51 cm (62nd centile, +0.32 SD), and a head circumference of 35.2 cm (38th centile, −0.31 SD). Clinical examination revealed a dysplastic left ear, a dysplastic left iliac wing, an asymmetrical face, plagiocephaly, hemifacial microsomia, hypermetropia, asymmetric crying facies (ACF), a preauricular pit in the right ear, right facial nerve paresis, hypotonia, developmental delay, weakness of the left muscles, and a dysplastic left lower jawbone. An abdomen and heart u/s produced normal results. A brain CT and brain MRI also produced normal results. However, a brain ultrasound revealed a cyst in the transparent septum (5.8 mm) as well as the left choroidal plexus (2.9 mm).

#### 2.2.12. Patient 12

Patient 12 is a 7-year-old boy who presented with developmental delay and autism spectrum disorder. His phenotypic features were mild, as he was able to attend regular school with occasional additional support. He did not exhibit any dysmorphic features.

#### 2.2.13. Patients 13 and 14

Patients 13 and 14 are 4-year-old male monozygotic twins. They presented with similar clinical features that included the absence of speech, autism spectrum disorder, stereotypic behavior, obsessions, and obesity. They first walked at the age of 18 months.

#### 2.2.14. Patient 15

Patient 15 is a 13-year-old girl, the half-sister from the mother’s side of the previous twin patients who presented with mild intellectual disability and autism spectrum disorder. She presented with seizures which were resolved by the age of 2. She is attending a special school. She did not have any dysmorphic features.

### 2.3. Methods

#### 2.3.1. Isolation of Genomic DNA

Genomic DNA was extracted from peripheral blood lymphocytes using a commercially available QiAmp DNA Blood Mini kit (Qiagen, Hilden, Germany), according to the manufacturer’s instructions. The quality and quantity of the DNA samples were determined using a NanoDrop ND-1000 UV-Vis spectrophotometer (Thermo Fisher Scientific, Waltham, MA, USA).

#### 2.3.2. Chromosomal Microarray Analysis—Array Comparative Genomic Hybridization

High-resolution aCGH analysis was performed on the DNA samples with the Agilent Human Genome 4 × 180 k CGH+SNP microarray platform, which offers ~120,000 CGH probes with a median probe spacing of ~25 Kb, as well as ~60,000 SNP probes that provide detection of Copy Neutral Loss of Heterozygosity (CN-LOH) with a resolution of ~5–10 Mb (Agilent Technologies, Santa Clara, CA, USA).

The protocol used for aCGH and analysis of the resulting data was as previously described [7,8], with CytoGenomics version 5.0.2 software (Agilent Technologies, Santa Clara, CA, USA) being used for both feature extraction and data analysis. The ADM-1 aberration detection method was utilized with a log2ratio threshold of 0.25 for both the deletions and duplications, with the minimum number of probes required for a call set to 4. The aberrations flagged by the software were also manually inspected to eliminate any false positive calls. For the location of genes in the deleted or duplicated genomic segments, the UCSC genome browser (http://genome.ucsc.edu/, accessed on 1 April 2024) and the Database of Genomic Variants (http://projects.tcag.ca/variation/, accessed on 1 April 2024; human genome build 19) were used.

Informed consent was obtained from the patients and/or their legal guardians prior to their enrollment in the study, which was conducted in accordance with the guidelines of the National and Kapodistrian University of Athens Bioethics Committee.

## 3. Results

A total of 13 deletions and two duplications of the 16p11.2 region were identified (Table 1), accounting for ~1% of all samples referred to the Laboratory of Medical Genetics for array CGH analysis from 2008 to 2023.

Eight deletions included the proximal 16p11.2 region (BP4–BP5) while five included the distal 16p11.2 region (BP2–BP3). Out of the two duplications that were identified, one affected the proximal region (BP4-BP5), and one affected the distal 16p11.2 region (BP2–BP3).

A high degree of phenotypic variability was observed between patients with the same aberration, ranging from mild neurodevelopmental features to profound speech delay and multiple congenital anomalies.

Patient 3, patient 5, patient 7, patient 10, patient 12, patient 13, patient 14, and patient 15 all presented with some form of developmental delay, while patient 10, patient 12, patient 13, patient 14, and patient 15 were additionally diagnosed with autism spectrum disorder. All patients also presented with mild to severe speech and language delays. Patient 3 and the twins, patient 13 and patient 14, presented with obesity, which is a common feature in proximal 16p11.2 deletion syndrome. Interestingly, these specific patients inherited microdeletion from their mother, who showed none of the hallmark features of the syndrome, highlighting the reduced penetrance associated with 16p11.2 rearrangements. Patient 3, patient 5, and patient 10 had normal brain MRI results.

Patient 3 and patient 10 were born at term with normal birth weights and growth metrics, patient 5 was born at 38 weeks with a lower birth weight, and patient 7 was born at term but significantly underweight (<1st centile).

Patient 3 has bilateral cryptorchidism and an arched palate, patient 5 has brachydactyly, vertebral anomaly, a club foot, scoliosis, lordosis, and a hypoplastic ilium, patient 7 has hypermetropia, a small nose, a small jaw, a pointy chin, and autoimmune neutropenia, patient 10 has iris coloboma, optical nerve coloboma, strabismus, an atrial septal defect, and epilepsy, while patient 15 also presented with epilepsy and intellectual disability along with ASD. Patient 3 had recurrent respiratory infections and atopic dermatitis, while patient 7 had autoimmune neutropenia and was susceptible to infections.

Patient 4 had a duplication of the proximal region and presented with multiple congenital anomalies, not commonly associated with recurrent 16p11.2 CNVs. However, he additionally carried a 5 Mb duplication of 9p24.3–p24.1, as well as a 19.9Mb deletion of 10p15.3–p12.31 which contributed to their phenotypic severity. This imbalance is derived from a maternal balanced translocation between chromosomes 9 and 10 (46,XX,t(9;10)(p24;p13)).

Patients 2, 6, 8, 9, and 11 all had a deletion in the distal 16p11.2 region, between Breakpoints 2 and 3. All exhibited some form of developmental delay and psychomotor delay with varying degrees of severity. Patient 2, patient 6, and patient 9 had a low head circumference. However, patient 8 had a normal head circumference and patient 11 showed only mild microcephaly and craniofacial abnormalities. Patient 2, patient 6, patient 8, and patient 9 showed speech or language disorders of varying severity, with patient 6 using non-verbal communication or single words or syllables only.

With regard to birth weight and growth metrics, patient 2 and patient 11 were born at term with relatively normal birth weights and growth metrics, patient 6 was born at term with a slight growth deficit, patient 8 was born preterm at 36 weeks with a low birth weight, and patient 9 was born extremely preterm at 28 weeks with severe intrauterine growth restriction (IUGR).

Patient 6 presented with ASD, attention deficit, and stereotypic and obsessive behavior, patient 9 had significant neonatal complications including bronchopulmonary dysplasia, patent ductus arteriosus, a history of CMV infection, retinopathy, anemia, cryptorchidism, hypospadias, and icterus, and patient 11 had multiple dysmorphic features including a dysplastic ear, an iliac wing, an asymmetrical face, plagiocephaly, hemifacial microsomia, and facial nerve paresis. Also, he presented with hypermetropia and asymmetric crying facies.

Patient 9’s severe complications, such as bronchopulmonary dysplasia and retinopathy, linked to extreme prematurity and intrauterine growth restriction (IUGR), are less commonly documented in association with distal 16p11.2 deletion, although preterm birth and its complications can exacerbate the clinical picture. Of note, the deletion in this patient was inherited from his non-affected father.

Patient 11’s multiple dysmorphic features, such as hemifacial microsomia and a dysplastic jaw, add to the variability seen in phenotypic presentations of the syndrome, emphasizing that while certain features are common, the full clinical spectrum can vary widely.

Patient 1 had a duplication of the distal 16p11.2 region and presented with microcephaly, dysmorphic features, hypotonia, seizures, and psychomotor delay. The severity of the phenotype could be attributed to an additional 10.3 Mb deletion of the 1p36.33–p36.22 chromosomal region that was also identified in this specific patient.

Patients 13 and 14 (twins) and patient 15 had inherited the deletion from their phenotypically healthy mother.

## 4. Discussion

The 16p11.2 chromosomal region in humans harbors multiple segmental duplication loci which act as substrates for the formation of recurrent CNVs through NAHR. Although rare, 16p11.2 recurrent rearrangements are one of the most common genetic causes of ASD, accounting for about 0.6–1% of all cases [9,10,11]. Additionally, they show a wide spectrum of other clinical manifestations ranging from intellectual disabilities and neuropsychiatric disorders with or without congenital anomalies, obesity, and learning and speech difficulties to normal phenotypes [12]. This phenotypic variability and/or reduced penetrance leads to highly discordant phenotypes even in patients from the same family, making the interpretation of these findings very difficult, especially in the prenatal setting. Possible explanations for this variability include rare or common genetic variants elsewhere in the genome that act as modifying factors, the unmasking of a heterozygous mutation by microdeletion in one of the genes in the region, as well as epigenetic and environmental influences.

Here, we reported on 15 patients with recurrent 16p11.2 rearrangements, aiming to further delineate the phenotypic spectrum of the specific aberrations. Overall, in our cohort, 6/15 patients had an aberration in the distal region of 16p11.2 between Breakpoints 2 and 3 (deletion: patients 3, 5, 7, 10,12, 13, and 14, duplication: patient 1) and 9/15 patients had an aberration in the proximal region (BP4-BP5) (deletion: patients 2, 6, 8, 9, and 11, duplication: patient 4).

The most common and well-characterized 16p11.2 CNVs affect the proximal region, leading to a ~593 Kb reciprocal deletion or duplication (BP4-BP5). Patients with the proximal deletion exhibit intellectual disability and speech impairment (70%), motor coordination difficulties (60%), ASD (20–25%), and seizures [13]. Deletions and duplications of the 16p11.2 BP4-BP5 region exhibit a “mirror effect”, mostly apparent in head circumference, with macrocephaly and microcephaly being observed, respectively [14]. The deletion is also associated with obesity, while the duplication carriers exhibit low weight. These mirror phenotypes are a possible indication of a gene-dosage effect for patients with recurrent 16p11.2 rearrangements. A recent study also suggested that atypical neural variability identified through EEG on 16p11.2 CNV carriers could also be indicative of the different effects of gene dosage [15]. Interestingly, however, while most patients carrying 593 Kb microdeletions tend to be overweight, there have been recent reports of patients with poor height and weight development [16], indicative of the complicated mechanisms involved in the manifestation of the clinical phenotype. The most important genes included in the region are *MAPK3*, *PRRT2*, *TBX6*, *ALDOA*, *CORO1A*, *MAZ*, *TAOK2*, and *KIF22*. Of these, *CORO1A*, *TAOK2*, and *MAZ* are predicted to be haploinsufficient, thus possibly contributing to the phenotypic features of deletion carriers. *TBX6* has been associated with vertebral malformations and *PRRT2* has been associated with epilepsy and paroxysmal dyskinesia. Another gene with potential contribution to the ASD and macrocephaly phenotypes is *KCTD13* [13].

Reduced head circumference in duplication carriers can lead to problems with brain development during the embryonic stage due to reduced overall cortical thickness, as well as various subcortical structures [17]. A study by McCarthy and colleagues found a strong association between 16p11.2 duplication and psychiatric disorders, mainly schizophrenia, bipolar disorder, and autism [18]. In contrast, patients with 16p11.2 microdeletion mainly exhibited autism and developmental disorders, enhancing the notion that clinical features differ between microdeletion and microduplication patients. Overall, duplication carriers exhibit less severe clinical features. The duplication is inherited in over 70% of the cases [13].

CNVs affecting the BP2-BP3 distal 16p11.2 region are less frequently observed. Similarly to proximal deletion, deletion of the distal region is associated with impaired cognitive ability presenting with incomplete penetrance, as well as obesity, macrocephaly, developmental delay, schizophrenia, and ASD [19]. Duplication carriers usually show a milder phenotype with mirror phenotypes similar to the ones affecting the proximal region. The most important genes included in this region are *SH2B1*, *TUFM*, *ATP2A1*, and *ATXN2L*; however, their exact function is not yet fully understood. *SH2B1* has been associated with the obesity phenotype and *ATXNL2* could play a role in central nervous system malfunction [19].

Microdeletions in the 16p11.2 region that include the *SH2B1* gene have been implicated as risk factors for IUGR, congenital heart anomalies, dysmorphic features, and developmental delay, although with incomplete penetrance. This has been underlined by a recent report on monozygotic twin boys that carried the same 244 Kb 16p11.2 microdeletion that included the *SH2B1* gene, which revealed discordant phenotypes for the two infants, with one carrying the hallmark features while the other showed a normal phenotype [20].

## 5. Conclusions

Our clinical and molecular findings support the described distinct phenotypic consequences of CNVs affecting the 16p11.2 region, as well as the marked variability in clinical severity observed even in patients within the same family. Regarding the patients in our cohort carrying duplications, the more severe phenotypic presentation that they exhibit (i.e., speech delay and dysmorphic features) can be attributed to the additional chromosomal imbalances they carry (Table 1).

A rarely discussed aspect of care for patients with rare genetic syndromes is the lack of knowledge observed among professionals, coupled with the high degree of phenotypic variability that many of these syndromes exhibit, leading to subpar medical care and socio-educational support [21]. As the symptoms are mostly non-indicative, a diagnosis can not be ascertained based on clinical evaluation alone and thus genetic testing should be offered to all patients with similar symptoms, including obesity, in order to identify the patients with 16p11.2 and provide timely individualized care.

In this study, we have provided a report on the phenotypic manifestations of 15 patients with recurrent 16p11.2 chromosomal rearrangements, aiming to further delineate the clinical spectrum. This information can provide an important reference to aid the counseling of patients and their families. Further research on the function of the genes involved in the rearranged segments as well as their interaction with other genomic loci will be required to elucidate the complex mechanisms underlying the variable expressivity exhibited by 16p11.2 recurrent rearrangements.

## Figures and Tables

**Table 1:** Molecular findings and clinical features of patients with 16p11.2 recurrent rearrangements.

Patient	Gender	Age at Diagnosis	Genotype/Size	Additional Findings	Breakpoints	Genes Included in the 16p11.2 Region	Clinical Summary
1	F	5 months	arr[GRCh37] 16p11.2(28629244–29031059) ×3 dn/402 Kb	arr[GRCh37] 1p36.33p36.22(564424–10892722) ×1dn/10.3 Mb	BP2–BP3 (Distal)	*SULT1A1*, *EIF3C*, *EIF3CL*, *ATXN2L*, *TUFM*, *SH2B1*, *ATP2A1*, *RABEP2*, *CD19*, *NFATC2IP*, *SPNS1*, *LAT*	Microcephaly, peculiar face, hypotonia, seizures since 1 month of age, brain MRI dysplastic right hemisphere with smaller dimensions, and pachygyria. At age 6 she does not walk and can only eat mashed foods
2	F	1 year	arr[GRCh37] 16p11.2(28732295–28949619) ×1/220 Kb		BP2–BP3 (Distal)	*ATXN2L*, *TUFM*, *SH2B1*, *ATP2A1*, *RABEP2*, *CD19*, *NFATC2IP*, *SPNS1*, *LAT*	Microcephaly (3rd centile), psychomotor delay, brain MRI increased T2 signal and flair images
3	M	6 years	arr[GRCh37] 16p11.2(29652999–30198600) ×1/545 Kb		BP4–BP5 (Proximal)	*SPN*, *QPRT*, *C16orf54*, *ZG16*, *KIF22*, *MAZ*, *PRRT2*, *C16orf53*, *MVP*, *CDIPT*, *LOC440356*, *SEZ6L2*, *ASPHD1*, *KCTD13*, *TMEM219*, *TAOK2*, *HIRIP3*, *INO80E*, *DOC2A*, *C16orf92*, *FAM57B*, *ALDOA*, *PPP4C*, *TBX6*, *YPEL3*, *GDPD3*, *MAPK3*, *LOC100271831*, *CORO1A*	Flat forehead, flat face, deep-set eyes, cryptorchidism, hypotonia, psychomotor delay, obesity, and speech delay
4	F	6 months	arr[GRCh37] 16p11.2(29652999–30197341) ×3/545 Kb	arr[GRCh37] 9p24.3p24.1(204193–5162606) ×1/4.96 Mbarr[GRCh37] 10p15.3p12.31(136361–20023236) ×3/19.9 Mb	BP4–BP5 (Proximal)	Same as above	36 cm occipitofrontal circumference, 52 cm length, deep-set eyes, seizures, sandal gap, heart U/S ventricular septal defect, enlarged left lateral abdomen. Prenatally hydronephrosis, renal cyst, and hypoglycemia
5	M	15 years	arr[GRCh37] 16p11.2(29652999–30198600) ×1/545 Kb		BP4–BP5 (Proximal)	Same as above	Epilepsy, seizures, short stature, brachydactyly, scoliosis, normal OCF circumference, and learning difficulties
6	M	5 years	arr[GRCh37] 16p11.2(28824794–29031059) ×1/206 Kb		BP2–BP3 (Distal)	*ATXN2L*, *TUFM*, *MIR4721*, *SH2B1*, *ATP2A1*, *ATP2A1-AS1*, *RABEP2*, *CD19*, *NFATC2IP*, *MIR4517*, *SPNS1*, *LAT*	Profound speech delay, autism, stereotypic behavior, obsessions, and obesity
7	M	4 years	arr[GRCh37] 16p11.2(29592783–30190568) ×1/598 Kb		BP4–BP5 (Proximal)	*SMG1P2*, *MIR3680-2*, *MIR3680-1*, *SPN*, *QPRT*, *C16orf54*, *ZG16*, *KIF22*, *MAZ*, *PRRT2*, *PAGR1*, *MVP*, *CDIPT*, *CDIPT-AS1*, *SEZ6L2*, *ASPHD1*, *KCTD13*, *TMEM219*, *TAOK2*, *HIRIP3*, *INO80E*, *DOC2A*, *C16orf92*, *FAM57B*, *ALDOA*, *PPP4C*, *TBX6*, *YPEL3*, *LOC101928595*, *GDPD3*, *MAPK3*	Macrocephaly, ID, IVF twin, hyperopia, hyperactivity, speech delay, neurodevelopmental disorder, autoimmune neutropenia, and allergies
8	M	4 years	arr[GRCh37] 16p11.2(28824794–29031059) ×1/206 Kb		BP2–BP3 (Distal)	*ATXN2L*, *TUFM*, *MIR4721*, *SH2B1*, *ATP2A1*, *ATP2A1-AS1*, *RABEP2*, *CD19*, *NFATC2IP*, *MIR4517*, *SPNS1*, *LAT*	Normal OCF circumference, psychomotor delay, and global developmental delay
9	M	3 years	arr[GRCh37] 16p11.2(28503803–29182196) ×1 pat/678 Kb		BP2–BP3 (Distal)	*APOBR*, *IL27*, *NUPR1*, *SGF29*, *SULT1A2*, *SULT1A1*, *NPIPB8*, *EIF3C*, *EIF3CL*, *MIR6862-1*, *MIR6862-2*, *NPIPB9*, *ATXN2L*, *TUFM*, *MIR4721*, *SH2B1*, *ATP2A1*, *ATP2A1-AS1*, *RABEP2*, *CD19*, *NFATC2IP*, *MIR4517*, *SPNS1*, *LAT*, *RRN3P2*	Microcephaly, IUGR, speech delay, developmental delay, flat face, broad forehead, cryptorchidism, and hypospadias inherited from unaffected father
10	M	3 years	arr[GRCh37] 16p11.2(29572030–30106101) ×1/530 Kb		BP4–BP5 (Proximal)	*SPN*, *QPRT*, *C16orf54*, *ZG16*, *KIF22*, *MAZ*, *PRRT2*, *PAGR1*, *MVP*, *CDIPT*, *CDIPTOSP*, *SEZ6L2*, *ASPHD1*, *KCTD13*, *TMEM219*, *TAOK2*, *HIRIP3*, *INO80E*, *DOC2A*, *C16orf92*, *TLCD3B*, *LOC112694756*, *ALDOA*, *PPP4C*, *TBX6*, *YPEL3*, *LOC101928595*, *GDPD3*, *MAPK3*	Microcephaly, round face, flat face, sparse eyebrows, strabismus, incident of seizures, developmental delay, iris coloboma, and optical nerve coloboma
11	M	1 year	arr[GRCh37] 16p11.2(28824794–29031059) ×1/206 Kb		BP2–BP3 (Distal)	*ATXN2L*, *TUFM*, *MIR4721*, *SH2B1*, *ATP2A1*, *ATP2A1-AS1*, *RABEP2*, *CD19*, *NFATC2IP*, *MIR4517*, *SPNS1*, *LAT*	Facial asymmetry, dysplastic ear, unilateral hearing loss, hyperopia, and developmental delay
12	M	7 years	arr[GRCh37] 16p11.2(29656684–30190568) ×1/534 Kb		BP4–BP5 (Proximal)	*SPN*, *QPRT*, *C16orf54*, *ZG16*, *KIF22*, *MAZ*, *PRRT2*, *PAGR1*, *MVP*, *CDIPT*, *CDIPTOSP*, *SEZ6L2*, *ASPHD1*, *KCTD13*, *TMEM219*, *TAOK2*, *HIRIP3*, *INO80E*, *DOC2A*, *C16orf92*, *TLCD3B*, *LOC112694756*, *ALDOA*, *PPP4C*, *TBX6*, *YPEL3*, *LOC101928595*, *GDPD3*, *MAPK3*	Developmental delay, ASD, attending regular school with occasional additional help, and no dysmorphic features
13	M	4 years	arr[GRCh37] 16p11.2(29656684–30190568) ×1 mat/534 Kb		BP4–BP5 (Proximal)	Same as above	Profound speech delay, autism, stereotypic behavior, obsessions, and obesity inherited from unaffected mother
14	M	4 years	arr[GRCh37] 16p11.2(29656684–30190568) ×1 mat/534 Kb		BP4–BP5 (Proximal)	Same as above	Profound speech delay, autism, stereotypic behavior, obsessions, and obesity inherited from unaffected mother
15	F	13 years	arr[GRCh37] 16p11.2(29656684–30190568) ×1 mat/534 Kb		BP4–BP5 (Proximal)	Same as above	Intellectual disability, epilepsy, and autism inherited from unaffected mother

## Data Availability

The data presented in this study are available on request from the corresponding author due to privacy or ethical restrictions.

## References

[1] Bachmann-Gagescu R., Mefford H.C., Cowan C., Glew G.M., Hing A.V., Wallace S., Bader P.I., Hamati A., Reitnauer P.J., Smith R. (2010). Recurrent 200-kb deletions of 16p11.2 that include the SH2B1 gene are associated with developmental delay and obesity. Genet. Med..

[2] Dittwald P., Gambin T., Szafranski P., Li J., Amato S., Divon M.Y., Rojas L.X.R., Elton L.E., Scott D.A., Schaaf C.P. (2013). NAHR-mediated copy-number variants in a clinical population: Mechanistic insights into both genomic disorders and Mendelizing traits. Genome Res..

[3] Sønderby I.E., Gústafsson Ó., Doan N.T., Hibar D.P., Martin-Brevet S., Abdellaoui A., Ames D., Amunts K., Andersson M., Armstrong N.J. (2018). Dose response of the 16p11.2 distal copy number variant on intracranial volume and basal ganglia. Mol. Psychiatry.

[4] Bochukova E.G., Huang N., Keogh J., Henning E., Purmann C., Blaszczyk K., Saeed S., Hamilton-Shield J., Clayton-Smith J., O’rahilly S. (2010). Large, rare chromosomal deletions associated with severe early-onset obesity. Nature.

[5] Schaaf C.P., Goin-Kochel R.P., Nowell K.P., Hunter J.V., Aleck K.A., Cox S., Patel A., Bacino C.A., Shinawi M. (2011). Expanding the clinical spectrum of the 16p11.2 chromosomal rearrangements: Three patients with syringomyelia. Eur. J. Hum. Genet..

[6] Poot M. (2018). Syndromes Hidden within the 16p11.2 Deletion Region. Mol. Syndromol..

[7] Tzetis M., Kitsiou-Tzeli S., Frysira H., Xaidara A., Kanavakis E. (2012). The clinical utility of molecular karyotyping using high-resolution array-comparative genomic hybridization. Expert Rev. Mol. Diagn..

[8] Oikonomakis V., Kosma K., Mitrakos A., Sofocleous C., Pervanidou P., Syrmou A., Pampanos A., Psoni S., Fryssira H., Kanavakis E. (2016). Recurrent copy number variations as risk factors for autism spectrum disorders: Analysis of the clinical implications. Clin. Genet..

[9] Kumar R.A., Karamohamed S., Sudi J., Conrad D.F., Brune C., Badner J.A., Gilliam T.C., Nowak N.J., Cook E.H., Dobyns W.B. (2008). Recurrent 16p11.2 microdeletions in autism. Hum. Mol. Genet..

[10] Weiss L.A., Shen Y., Korn J.M., Arking D.E., Miller D.T., Fossdal R., Saemundsen E., Stefansson H., Ferreira M.A.R., Green T. (2008). Association between Microdeletion and Microduplication at 16p11.2 and Autism. N. Engl. J. Med..

[11] Sanders S.J., He X., Willsey A.J., Ercan-Sencicek A.G., Samocha K.E., Cicek A.E., Murtha M.T., Bal V.H., Bishop S.L., Dong S. (2015). Insights into Autism Spectrum Disorder Genomic Architecture and Biology from 71 Risk Loci. Neuron.

[12] Bijlsma E.K., Gijsbers A.C.J., Schuurs-Hoeijmakers J.H.M., van Haeringen A., Fransen van de Putte D.E., Anderlid B.M., Lundin J., Lapunzina P., Jurado L.A.P., Chiaie B.D. (2009). Extending the phenotype of recurrent rearrangements of 16p11.2: Deletions in mentally retarded patients without autism and in normal individuals. Eur. J. Med. Genet..

[13] Chung W.K., Roberts T.P., Sherr E.H., Snyder L.A.G., Spiro J.E. (2021). 16p11.2 deletion syndrome. Curr. Opin. Genet. Dev..

[14] Cárdenas-de-la-Parra A., Martin-Brevet S., Moreau C., Rodriguez-Herreros B., Fonov V.S., Maillard A.M., Zürcher N.R., Hadjikhani N., Beckmann J.S., 16p11.2 European Consortium (2019). Developmental trajectories of neuroanatomical alterations associated with the 16p11.2 Copy Number Variations. Neuroimage.

[15] Al-Jawahiri R., Jones M., Milne E. (2019). Atypical neural variability in carriers of 16p11.2 copy number variants. Autism Res..

[16] Dell’Edera D., Dilucca C., Allegretti A., Simone F., Lupo M.G., Liccese C., Davanzo R. (2018). 16p11.2 microdeletion syndrome: A case report. J. Med. Case Rep..

[17] Qureshi A.Y., Mueller S., Snyder A.Z., Mukherjee P., Berman J.I., Roberts T.P.L., Nagarajan S.S., Spiro J.E., Chung W.K., Sherr E.H. (2014). Opposing brain differences in 16p11.2 deletion and duplication carriers. J. Neurosci..

[18] McCarthy S.E., Makarov V., Kirov G., Addington A.M., McClellan J., Yoon S., Perkins D.O., Dickel D.E., Kusenda M., Krastoshevsky O. (2009). Microduplications of 16p11.2 are associated with schizophrenia. Nat. Genet..

[19] Woodbury-Smith M., D’Abate L., Stavropoulos D.J., Howe J., Drmic I., Hoang N., Zarrei M., Trost B., Iaboni A., Anagnostou E. (2023). The Phenotypic variability of 16p11.2 distal BP2-BP3 deletion in a transgenerational family and in neurodevelopmentally ascertained samples. J. Med. Genet..

[20] Li L., Huang L., Lin S., Luo Y., Fang Q. (2017). Discordant phenotypes in monozygotic twins with 16p11.2 microdeletions including the SH2B1 gene. Am. J. Med. Genet. A.

[21] Kleinendorst L., van den Heuvel L.M., Henneman L., van Haelst M.M. (2020). Who ever heard of 16p11.2 deletion syndrome? Parents’ perspectives on a susceptibility copy number variation syndrome. Eur. J. Hum. Genet..

